# Comparative Analysis of Erythrocyte Proteomes of Water Buffalo, Dairy Cattle, and Beef Cattle by Shotgun LC-MS/MS

**DOI:** 10.3389/fvets.2019.00346

**Published:** 2019-10-18

**Authors:** Jiaying Guo, Yali Sun, Yu Tian, Junlong Zhao

**Affiliations:** ^1^State Key Laboratory of Agricultural Microbiology, College of Veterinary Medicine, Huazhong Agricultural University, Wuhan, China; ^2^Key Laboratory of Development of Veterinary Diagnostic Products, Ministry of Agriculture of the People's Republic of China, Wuhan, China; ^3^Key Laboratory of Animal Epidemical Disease and Infectious Zoonoses, Ministry of Agriculture, Huazhong Agricultural University, Wuhan, China

**Keywords:** *Babesia orientalis*, water buffalo, dairy cattle, beef cattle, erythrocyte, proteome

## Abstract

A number of studies have demonstrated that *Babesia orientalis* (*B. orientalis*) can only infect water buffalo (*Bubalus bubalis*) and not dairy cattle (*Bos taurus*) or beef cattle (*Bos taurus*), even though all three belong to the tribe *Bovini* and have close evolutionary relationships. In addition, *Babesia* species are intracellular protozoans that obligately parasitize in erythrocytes. This may indicate that the infection specificity is due to differences in erythrocyte proteins. Totals of 491, 1,143, and 1,145 proteins were identified from water buffalo, beef cattle, and dairy cattle, respectively, by searching the Uniprot and NCBI databases. The number of proteins identified for water buffalo was far lower than for beef cattle and dairy cattle, particularly in the range from 15 to 25 kDa. Remarkably, 290 identified proteins were unique to water buffalo, of which putative gamma-globin and putative epsilon-globin had a significant possibility of being relevant to the survival of *B. orientalis* only in water buffalo. A total of 2,222 proteins were annotated in terms of molecular function, biological process, and cellular component according to GO annotation. The number of proteins of water buffalo in oxygen binding was far higher than for beef cattle and dairy cattle. This is the first time that the protein profiles of water buffalo, beef cattle, and dairy cattle have been comparatively analyzed. The uniquely expressed proteins in water buffalo obtained in this study may provide new insights into the mechanism of *B. orientalis* infection exclusivity in water buffalo and may be a benefit for the development of strategies against *B. orientalis*.

## Introduction

*Babesia* is a tick-borne apicomplexan parasite that can cause a zoonotic disease known as babesiosis (1–3). A unique characteristic of *Babesia* is that it is obligate to parasitize and reproduce within erythrocytes. It can infect an extensive range of mammalian and even humans. The main clinical presentations are fever, anemia, hemoglobinuria, jaundice, and even death (2, 4). *Babesia* gives rise to a massive burden of morbidity, which not only leads to enormous economic losses but also hampers the development of the livestock industry (1, 2, 4, 5). Over 100 species of *Babesia* have been reported, and they have a worldwide distribution (1). In China, five of these can infect cattle, namely *Babesia bovis* (*B. bovis*), *Babesia bigemina* (*B. bigemina*), *Babesia ovata, Babesia major*, and *Babesia orientalis* (*B. orientalis*) (4). *B. orientalis* was first discovered in central and south China in 1984 (4, 6, 7). According to the pathogenicity, morphology, *in vitro* cultivation characteristics, and phylogenetic analysis of the 18S rRNA gene, it was recognized as a new *Babesia* species and named *B. orientalis* in 1997 (4, 8–12). Furthermore, it is only transmitted by *Rhipicephalus haemaphysaloides* and exclusively parasitizes in the erythrocytes of water buffalo rather than in those of beef cattle or dairy cattle (1, 4, 10, 13–15). In contrast, *B. bovis* and *B. bigemina* can infect both water buffalo and cattle through *Rhipicephalus* and *Ixodes* (1). Even though substantial efforts have been made in genome sequencing, *in vitro* cultivation, and with diagnostic methods, the molecular mechanism of the specific invasion of the erythrocytes of water buffalo remains unknown (13, 16, 17). In terms of genome sequencing, only the mitochondrial and apicoplast genomes of *B. orientalis* and the whole genome of water buffalo have been reported, which provide little information clarifying the mechanisms of invasion specificity (18, 19).

Proteomics, an emerging technology, refers to the protein-expression profiles of a gene, a cell, or a tissue in a particular period (5, 20–22). Unlike the immutable genomics, proteomics is a post-genomic method and is preferentially sensitive to dynamic changes in the parasite and the host; that is, it changes along with the environment. An increasing number of proteomic methods are used to determine the differences between normal and diseased states so as to search for potential drugs and treatment targets (21, 23). For instance, proteomics is used in the analysis of female *Rhipicephalus Microplus*-stage-specific protein expression of *B. bovis* and also in finding biomarkers for the diagnosis of *Babesia canis* (21, 24). In addition, a protein profile of mammalian erythrocyte membranes has been identified by matrix-assisted laser desorption/ionization time-of-flight/mass spectrometer (MALDI-TOF/MS) (22). Proteomics has also been applied to develop vaccines against tick-borne diseases (5). However, no reports have been made of the application of proteomics to *B. orientalis*, and no comparison has been made of the erythrocyte proteins of water buffalo, beef cattle, and dairy cattle. There are several approaches to the study of proteomics, such as by two-dimensional electrophoresis/mass spectrometer (2-DE/MS), MALDI-TOF/MS, or liquid chromatography mass spectrometer (LC-MS/MS) (25–28). The shotgun method has the advantage of identifying more proteins than other methods of proteomics, including proteins that have extreme isoelectric point (pI) and molecular mass (Mw) values (26, 29, 30).

To clarify the mechanism of infection specificity, three aspects should be considered: the host, the parasite, and both in conjunction. As a result, this article focuses on the host: the erythrocyte of water buffalo. In this study, proteomics were used to find differences among the erythrocytes of water buffalo, beef cattle, and dairy cattle, which may provide new insights into the mechanisms by which *B. orientalis* exclusively parasitizes the erythrocytes of water buffalo and may be beneficial for devising strategies for inhibiting the survival and replication of *B. orientalis* in those erythrocytes.

## Materials and Methods

### Experimental Animals and Blood Collection

A 1-year-old water buffalo, 1-year-old beef cattle and 1-year-old dairy cattle were verified free of *B. orientalis* by microscopic examination, reverse line blot, and real-time PCR (13, 17). All of the blood samples were withdraw into sterile vacuum tubes containing anticoagulant with EDTA (1.5 mg/ml blood).

### Erythrocyte Protein Preparation

The steps taken to purify the red blood cells (RBCs) were essential and indispensable for LC-MS/MS. The procedures used in this article followed the previously reported protocols of Pesciotta et al. (31), Pasini et al. (32), and Bryk and Wisniewski (28) combined. In brief, the small cells, such as platelets and microparticles, were removed by low-speed centrifuge and multiple cold buffer washes. RBCs were centrifuged at 1,500 rpm for 10 min at 4 °C. The supernatant, especially the layer of white blood cells, was removed. The pellet was resuspended in cold phosphate-buffered saline (PBS) to the original volume and then mixed gently. The above steps were repeated three times or more until the supernatant was clean. Even though most of the white blood cells had been discarded by removing the layer of white blood cells, the remaining white blood cells and other cells larger than RBCs needed to be removed by white cell filters (Plasmodipur, Euro-diagnostica, Arnhem, the Netherlands). The purification of the RBC pellet was evaluated by making smears, and the number of non-RBCs was counted by microscopy. Once the ratio of non-RBCs (the number of non-RBCS/total cells) was <0.001, the RBC pellet could be subjected to the next steps (31, 32).

Next, the RBC pellet was lysed with 20 ml of cold red cell lysis buffer (Tiangen Biotech, Beijing, China), standing for 30 min at 4°C. The RBCs were then beaten 15–20 times using a 1-ml syringe. The lysate was then centrifuged at 12,000 rpm for 10 min at 4°C. The pellet was suspended in 30 ml of PBS, and the above step was repeated five more times. Finally, the resuspended protein solution was stored in PBS at −20°C for 1-DE and shotgun analysis.

### SDS-PAGE and Silver Staining

One hundred milligrams of proteins from each specimen was denatured in an equal volume of 2 × protein loading buffer (0.2 M DTT, 20% glycerol, 0.1 M Tris-HCl, pH 6.8, 4% SDS, 0.2% bromophnol blue) at 100°C for 12 min. Denatured proteins were separated through 12% SDS-PAGE at 70 V for 30 min and then 100 V for 1 h. The gel was then stained for 30 min in a solution containing 0.07% (wt./vol.) coomassie brilliant blue G250 (CBB) (Invitrogen, Carlsbad, CA, USA). The SDS-PAGE gels were stained with a silver kit (Beyotime Biotechnology, Shanghai, China).

### Filter-Aided Sample Preparation (FASP Digestion)

Two hundred micrograms of proteins for each sample was incorporated into 30 μl of SDT buffer (4% SDS, 100 mM DTT, 150 mM Tris-HCl pH 8.0). The detergent, DTT, and other low-molecular-weight components were removed using UA buffer (8 M Urea, 150 mM Tris-HCl pH 8.0) by repeated ultrafiltration (Microcon units, 10 kD). Then, 100 μl of iodoacetamide (100 mM IAA in UA buffer) was added to block reduced cysteine residues, and the samples were incubated for 30 min in darkness. The filters were washed with 100 μl of UA buffer three times and then with 100 μl of 25 mM NH_4_HCO_3_ buffer twice. Finally, the protein suspensions were digested with 4 μg of trypsin (Promega, Madison, Wisconsin, US) in 40 μl of 25 mM NH_4_HCO_3_ buffer overnight at 37°C, and the resulting peptides were collected as a filtrate. The peptides of each sample were desalted on C18 Cartridges [Empore™ SPE Cartridges C18 (standard density)], bed I.D. 7 mm, volume 3 ml (Sigma), concentrated by vacuum centrifugation and reconstituted in 40 μl of 0.1% (v/v) formic acid. The peptide content was estimated by UV light spectral density at 280 nm using an extinction coefficient of 1.1 of 0.1% (g/l) solution, which was calculated on the basis of the frequency of tryptophan and tyrosine in vertebrate proteins.

### HPLC-ESI-MS/MS (Shotgun Analysis)

The peptide mixture (3 ug) was loaded onto a reversed-phase trap column (Thermo Scientific Acclaim PepMap100, 100 μm × 200 mm, nanoViper C18) connected to a C18 reversed-phase analytical column (Thermo Scientific Easy Column, 10 cm long, 75 μm inner diameter, 3 μm resin) in buffer A (0.1% Formic acid) and separated with a linear gradient of buffer B (84% acetonitrile and 0.1% Formic acid) at a flow rate of 300 nl/min controlled by IntelliFlow technology. The linear gradient was determined by the project proposal: 0–35% buffer B for 50 min, 35–100% buffer B for 5 min, then being held in 100% buffer B for 5 min.

LC-MS/MS analysis was performed on a Q Exactive mass spectrometer (Thermo Scientific) that was coupled to an Easy nLC (Thermo Fisher Scientific) for 60 min. The mass spectrometer was operated in positive ion mode. MS data were acquired using a data-dependent top 10 method, dynamically choosing the most abundant precursor ions from the survey scan (300–1,800 m/z) for HCD fragmentation. The automatic gain control (AGC) target was set to 3e6 and the maximum inject time to 10 ms. The dynamic exclusion duration was 40.0 s. Survey scans were acquired at a resolution of 70,000 at m/z 200, the resolution for HCD spectra was set to 17, 500 at m/z 200, and the isolation width was 2 m/z. The normalized collision energy was 30 eV, and the underfill ratio, which specifies the minimum percentage of the target value likely to be reached at maximum fill time, was defined as 0.1%. The instrument was run with peptide recognition mode enabled.

### Protein Identification and Annotation

The MS/MS spectra were searched for in the UniProtKB *Bovinae* and *Babesia* database (56445 total entries, downloaded 20170807) using the MASCOT engine (Matrix Science, London, UK; version 2.4). For protein identification, the following options were used. Peptide mass tolerance = 20 ppm, MS/MS tolerance = 0.1 Da, enzyme = trypsin, missed cleavage = 2, fixed modification: carbamidomethyl (C), variable modification:Oxidation (M), peptides FDR ≦ 0.01, protein FDR ≦ 0.01.

### Gene Ontology (GO) Annotation

The protein sequences of differentially expressed proteins were retrieved in batches from the UniProtKB database (UniProtKB *Bovinae* database). The retrieved sequences were locally searched for in the SwissProt database (UniProtKB *Bovinae* database) using NCBI BLAST+ client software to find homolog sequences from which the functional annotation could be transferred to the studied sequences. In this work, the top 10 blast hits with E-values of <1e-3 for each query sequence were retrieved and were loaded into Blast2GO9 (UniProtKB Bovinae database) for GO mapping and annotation. An annotation configuration with an E-value filter of 1e-6, default gradual EC weights, a GO weight of 5, and an annotation cutoff of 75 was chosen. Un-annotated sequences were then re-annotated with more permissive parameters. The sequences without BLAST hits and un-annotated sequences were then selected to go through an InterProScan10 against the EBI database to retrieve functional annotations of protein motifs, and the InterProScan GO terms were merged with the annotation set. The GO annotation results were plotted by R scripts.

## Results

### SDS-PAGE and Silver Staining

**Figure 1:**
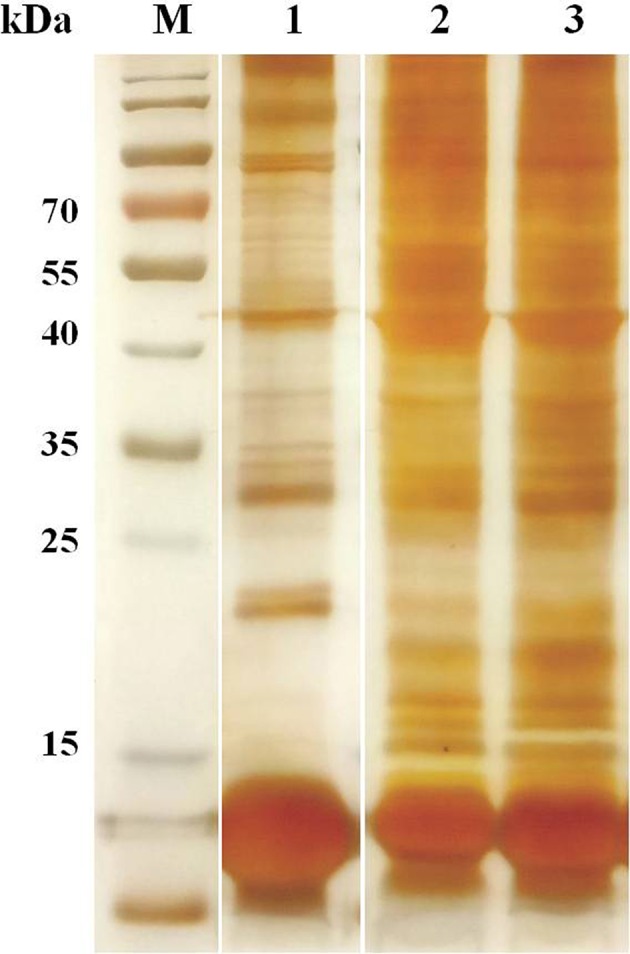
The erythrocyte proteins of water buffalo, beef cattle, and dairy cattle were separated by SDS-PAGE, and then silver staining was performed.

The erythrocyte proteins of water buffalo, beef cattle, and dairy cattle were separated by SDS-PAGE. The SDS-PAGE results were visualized through CBB and silver staining (Figure 1). There were obviously far more protein bands for beef and dairy cattle than for water buffalo, especially in the range from 15 to 25 kDa. The number of proteins detected by the shotgun method was obviously far higher than that through SDS-PAGE.

### Global Analysis of Erythrocyte Proteomes

Proteins were digested via FASP and were subjected to shotgun LC-ESI-MS/MS analysis. After removing redundant sequences, the identified proteins were searched for in the Uniprot and NCBI databases (Supplementary Table 1). A total of 491, 1,143, and 1,145 proteins (pepcount ≥1) were identified in water buffalo, beef cattle, and dairy cattle, with 4,012 peptides including 1,825 unique peptides, 6,771 peptides including 5,380 unique peptides, and 6,519 peptides including 4,881 unique peptides, respectively.

**Figure 2:**
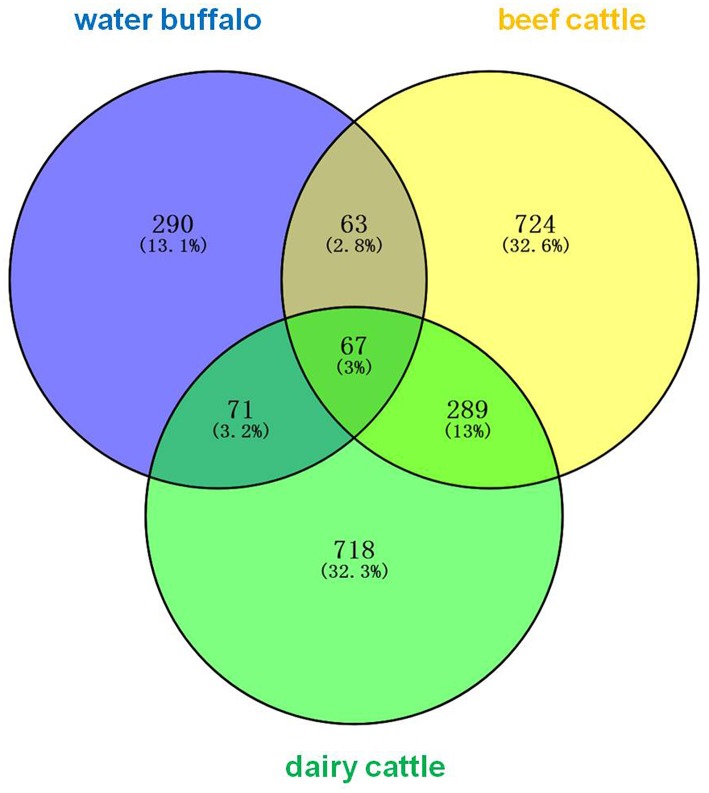
The erythrocyte proteins of water buffalo, beef cattle, and dairy cattle were identified and comparatively analyzed in a Venn diagram. The identified erythrocyte proteins of water buffalo are in blue, those of beef cattle are in yellow, and those of dairy cattle are in green.

The erythrocyte protein profiles of water buffalo, beef cattle, and dairy cattle were analyzed with the Venny 2.1.0 tool (http://bioinfogp.cnb.csic.es/tools/venny/index.html). The resulting Venn diagram is shown in Figure 2. It shows that 67 proteins were common to them all, the majority of which were house-keeping genes including ATP synthase subunits, heat shock proteins (HSP70 and HSP90), actin, tubulin, ribosomal protein, and so on. A total of 63 proteins were common to water buffalo and beef cattle, 71 proteins were common to water buffalo and dairy cattle, and 289 proteins were common to beef cattle and dairy cattle. In obvious contrast to water buffalo, the protein profile of beef cattle was, for the most part, relatively similar to that of dairy cattle. Furthermore, 290 proteins were water buffalo-biased, 718 proteins were dairy cattle-biased, and 724 proteins were beef cattle-biased. The 290 proteins that were only identified in water buffalo are detailed in Table 1; these might be related to the infection specificity.

**Table 1:** Proteins only identified in water buffalo.

**References**	**Name**	**PepCount**	**Unique PepCount**	**MW**	**PI**
G3MYA1	Uncharacterized protein	1	1	4457.3	9.85
Q9TS74	Pancreatic elastase inhibitor (Fragments)	1	1	6190.94	4.37
B1PC65	Testis-specific protein (Fragment)	1	1	7522.33	8.34
Q2NKR5	Chromosome 10 open reading frame 116 ortholog	2	2	7912.74	5.21
Q7M2Q9	Rho protein GDP-dissociation inhibitor (Fragments)	1	1	8077.95	4.43
L8I6H3	Small VCP/p97-interacting protein (Fragment)	1	1	8644.9	9.07
K9ZTI7	K-casein (Fragment)	1	1	9674.07	9.72
Q5XL27	Cathelicidin (Fragment)	1	1	10122.43	5.38
E1AHZ7	S100A8 protein	2	2	10381.8	5.12
L8HXY8	Histone H4	3	2	11367.2	11.36
F1MR08	SH3 domain-binding glutamic acid-rich-like protein	1	1	12471.91	5.78
Q2KII4	Elongin-C	1	1	12473.04	4.74
P63026	Vesicle-associated membrane protein 2	1	1	12648.56	7.84
L8I9D2	D-dopachrome decarboxylase	1	1	12876.81	6.59
E1BJ20	Uncharacterized protein	1	1	13195.79	4.87
L8HMV9	Hemoglobin fetal subunit beta (Fragment)	7	3	13221.97	7.15
Q32PA4	14 kDa phosphohistidine phosphatase	1	1	13930.38	5.49
L8IS67	Histone H2B	5	4	13936	10.31
L8II47	Nuclear transport factor 2	1	1	14478.33	5.1
B5A5S9	Fatty acid binding protein 4	1	1	14671.65	5.22
Q0II81	Regulator of G-protein signaling 13	1	1	14762.79	9.02
P04237	Hemoglobin subunit alpha	87	10	14948.83	8.22
Q56JX9	Fatty acid-binding protein, intestinal	1	1	15036.01	6.63
P55052	Fatty acid-binding protein, epidermal	1	1	15074.19	7.58
L8I0V6	Cytochrome b5	1	1	15328.86	4.94
L8IRE7	Histone H3	2	2	15403.91	11.13
L8HPK0	Protein S100-A9 (Fragment)	1	1	15405.93	5.75
L8HS15	Uncharacterized protein (Fragment)	5	5	15471.93	9.38
Q862Q0	Phosphoglycerate mutase (Fragment)	1	1	15518.4	7.92
Q52RN5	Superoxide dismutase [Cu-Zn]	4	2	15658.28	5.85
L8I1T9	Calcium-regulated heat stable protein 1	1	1	15890.78	7.74
D4QBF0	Hemoglobin beta	237	17	15986.2	6.7
D4QBF4	Hemoglobin beta	117	15	16006.23	7.06
A8E197	Adult beta-globin	194	16	16016.22	6.7
P04245	Hemoglobin subunit beta	120	15	16053.24	6.65
A8E1A0	Putative gamma-globin	130	14	16060.24	6.43
A8E199	Putative epsilon-globin	76	12	16106.3	6.49
A0A0A7NM42	Cathelicidin 4	1	1	16210.43	7.62
L8HUG2	Hemoglobin subunit epsilon-1 (Fragment)	6	2	16539.87	8.07
Q19RN7	Vimentin (Fragment)	1	1	17065.52	4.67
L8J1B2	Ubiquitin-conjugating enzyme E2 N	1	1	17137.61	6.13
Q45RQ8	Interferon-stimulated protein 17	3	3	17279.88	7.72
P52175	Nucleoside diphosphate kinase A 2	9	7	17297.84	7.77
P05630	ATP synthase subunit delta, mitochondrial	1	1	17611.86	5.2
L8IZ76	Uncharacterized protein (Fragment)	4	3	17810.53	4.23
A0A0A7UXB6	Cathelicidin 6	1	1	17882.7	9.51
Q32KU4	IQ domain-containing protein F5	1	1	18050.32	10.89
G3MZV0	Uncharacterized protein	3	3	18344.38	4.45
Q17QX0	Nudix (Nucleoside diphosphate linked moiety X)-type motif 5	3	3	18575.61	5.04
F1MGJ1	Uncharacterized protein	1	1	19785.34	9.39
L8IPP3	Protein DJ-1 OS = Bos mutus	2	2	20035.08	6.84
L8HVR1	Lactoylglutathione lyase (Fragment)	4	4	21067.72	5.09
Q29RM3	Receptor expression-enhancing protein 5	1	1	21416.68	8.27
L8IKY9	BH3-interacting domain death agonist (Fragment)	1	1	21421.95	5.46
L8IWW5	Heme-binding protein 1	1	1	21762.35	5.09
A7MAZ5	Histone H1.3	2	2	22153.36	10.97
P29104	Hippocalcin-like protein 4	1	1	22202.15	4.76
L8HSB9	Protein FADD	3	2	22246.14	6.59
L8IY35	Flavin reductase	7	3	22731.73	6.34
L8HVH6	Neutrophil gelatinase-associated lipocalin	1	1	22853.88	9.33
Q5GN72	Alpha-1-acid glycoprotein	1	1	23158.19	5.49
A4FV74	COPS8 protein	1	1	23201.31	5.25
Q2HJ25	Methionine aminopeptidase	1	1	23805.36	4.97
L8HNE8	Uncharacterized protein (Fragment)	1	1	24068.74	8.56
A5PK88	MGC159500 protein	4	4	24116.48	5.44
A8NJX6	DNAJA4 protein	1	1	24403.88	8.99
P02662	Alpha-S1-casein	2	2	24528.64	4.98
L8IJZ7	Mps one binder kinase activator-like 1A (Fragment)	3	3	24551.83	6.24
F1MZV2	Uncharacterized protein	1	1	24588.5	4.68
F1N3K8	Transmembrane emp24 domain-containing protein 10	1	1	25073.67	6.14
L8I6U5	Uncharacterized protein (Fragment)	4	4	25132.12	9.41
L8HPA7	Hypoxanthine-guanine phosphoribosyltransferase (Fragment)	1	1	25292.28	8.52
L8IZ09	HD domain-containing protein 2 (Fragment)	1	1	25346.56	5.67
A6QPC3	ADCK5 protein (Fragment)	1	1	25366.58	9.63
Q3MHN0	Proteasome subunit beta type-6	5	5	25541.77	4.9
E1BAB9	Uncharacterized protein	1	1	25732.03	4.22
Q37419	Cytochrome c oxidase subunit 2	1	1	26079.28	4.78
B6VPY2	Alpha s2 casein	1	1	26114.38	7.66
Q2TBG8	Ubiquitin carboxyl-terminal hydrolase isozyme L3	3	2	26181.27	4.84
P08166	Adenylate kinase 2, mitochondrial	1	1	26496.5	8.27
Q5E956	Triosephosphate isomerase	2	2	26689.19	6.45
Q0VCJ2	Methylthioribulose-1-phosphate dehydratase	1	1	27094.88	6.45
L8I399	Uncharacterized protein	2	2	27378.53	6.83
G3X760	Arginine and glutamate-rich protein 1	1	1	27388.29	10.54
Q2YDE4	Proteasome subunit alpha type-6	2	2	27399.16	6.35
P63103	14-3-3 protein zeta/delta	7	7	27744.79	4.73
L8IMN0	Cdc42 effector protein 3	1	1	27756.88	5.5
A6QL94	Izumo sperm-egg fusion protein 3	1	1	27840.92	8.98
L8I9I3	Lymphocyte function-associated antigen 3 (Fragment)	2	2	27990.78	5.06
F8UTU5	Catalase (Fragment)	9	7	28768.91	6.32
P47865	Aquaporin-1	2	2	28800.12	6.58
Q0VCU8	Eukaryotic translation initiation factor 3 subunit J	1	1	28950.87	4.72
L8I0A0	Carbonic anhydrase 2 (Fragment)	8	6	29342.77	6.2
L8I6V6	Uncharacterized protein (Fragment)	6	3	29462.47	6.2
L8IQH0	Proteasome subunit beta type	2	2	29937	6.9
Q3MHY8	RNA-binding protein 7	1	1	29962.58	9.66
A5PK91	LOC785621 protein	1	1	29974.84	6.42
Q3T014	Bisphosphoglycerate mutase	3	3	30060.91	6.03
L8J3F4	S-methyl-5'-thioadenosine phosphorylase (Fragment)	1	1	30222.66	6.82
L8HU80	COP9 signalosome complex subunit 7a	1	1	30299.27	8.34
A6H783	VDAC5P protein	3	3	30869.57	8.95
Q3T0T9	20-beta-hydroxysteroid dehydrogenase-like	1	1	31707.07	8.23
Q2HJ54	Phosphatidylinositol transfer protein alpha isoform	5	4	31849.06	6.12
Q32LE5	Isoaspartyl peptidase/L-asparaginase	1	1	32050.08	7
E1BNF9	Uncharacterized protein	1	1	32414.79	6.4
Q0P5E7	GTP-binding protein 8	1	1	32565.6	9.36
L8HVL1	L8HVL1_9CETA Ribose-5-phosphate isomerase	1	1	32682.06	8.96
F1MUL0	Leukocyte surface antigen CD47	1	1	33337.42	8.56
A5D7K0	Biliverdin reductase A	2	2	33643.33	5.85
Q6QRN7	PP1201 protein	2	2	33948.47	8.63
L8I8B2	Vacuolar protein sorting-associated protein VTA1-like protein	1	1	33966.9	5.87
B2BAV6	NAD(P)(+)–arginine ADP-ribosyltransferase (Fragment)	1	1	34067.97	9.31
Q32LM2	Small glutamine-rich tetratricopeptide repeat-containing protein alpha	2	2	34212.74	4.79
E1BMW9	Uncharacterized protein	1	1	34880.31	6.07
F1MK10	Uncharacterized protein	1	1	35030.63	7.78
L8IZ67	Glutaredoxin-3 (Fragment)	2	2	35101.93	5.85
L8HUU3	Olfactory receptor	1	1	35236.6	8.44
L8I9E8	KH domain-containing, RNA-binding, signal transduction-associated protein 3	1	1	35301	8.7
L8HYU9	Olfactory receptor	1	1	35328.73	8.82
E1BED0	Olfactory receptor	1	1	35360.03	8.76
L8IS42	Zeta-crystallin	7	5	35381.39	8.58
L8IGX7	Purine nucleoside phosphorylase (Fragment)	6	4	35395.99	6.34
G3N3W3	ADP/ATP translocase 2	4	4	35451.57	9.82
Q2HJB1	Vacuolar protein sorting 4 homolog A (S. cerevisiae)	1	1	35753.4	9.15
E1B9S2	Uncharacterized protein	1	1	36010.85	8.57
Q2KIL3	Delta-aminolevulinic acid dehydratase	7	7	36125.12	6.51
K0IT60	L-lactate dehydrogenase	6	6	36678.2	5.72
A0FH35	L-lactate dehydrogenase	6	6	36757.24	6.02
Q5E9I7	Methylosome protein 50	2	2	36789.01	5.18
G5E6P0	Uncharacterized protein	1	1	36974.06	8.87
L8HUZ2	Serine/threonine-protein phosphatase 2A activator (Fragment)	2	2	37650.81	6.03
F1N6P9	Uncharacterized protein	5	4	37845.22	8.96
B2BB07	Cluster of differentiation 2 (Fragment)	1	1	37852.22	9.21
A6QPX7	FGB protein (Fragment)	3	3	37931.09	6.29
L8ITF0	Ubiquitin thioesterase OTU1	1	1	38050.89	5.46
L8IGA2	Ubiquitin carboxyl-terminal hydrolase (Fragment)	1	1	38540.74	5.21
F1N650	Annexin	1	1	38979.24	6.38
L8IQW8	Guanine nucleotide-binding protein G(T) subunit alpha-1	1	1	39965.25	5.48
Q2YDD7	Galectin	1	1	40126.65	8.94
L8I9G4	Vasodilator-stimulated phosphoprotein (Fragment)	1	1	40403.61	8.78
L8IJV2	IST1-like protein (Fragment)	3	3	40596.87	5.16
L8HNS1	Ankyrin repeat and SAM domain-containing protein 4B (Fragment)	1	1	40979.12	4.93
L8I943	Tropomodulin-2	19	3	41376.62	5.38
Q58DA0	26S proteasome non-ATPase regulatory subunit 4	2	2	41383.92	4.68
L8IK86	Tubulin alpha chain (Fragment)	1	1	41792.25	4.69
L8HZA7	Phosphate carrier protein, mitochondrial (Fragment)	2	2	41897.45	9.47
L8INI8	Proteasomal ubiquitin receptor ADRM1	1	1	42014.54	4.8
Q148I1	Proteasomal ATPase-associated factor 1	1	1	42193.63	5.75
G1K1R6	Galactokinase	2	2	42242.49	5.77
Q5E964	26S proteasome non-ATPase regulatory subunit 13	4	4	42865.84	5.44
L8IHV3	26S proteasome non-ATPase regulatory subunit 11 (Fragment)	5	5	44237.58	6.4
L8J1T5	Protein DDI1-like protein 2	2	2	44469.07	4.98
Q3T0P6	Phosphoglycerate kinase 1	9	7	44537.11	8.48
L8INZ1	Fructose-bisphosphate aldolase	6	6	45108.08	8.66
L8IWP1	Zinc finger CCCH domain-containing protein 15 (Fragment)	1	1	45763.52	4.96
A4IFA6	Immunoglobulin superfamily containing leucine-rich repeat protein	1	1	45771.6	5.47
O97760	Rhesus-like protein	4	3	46024.08	9.23
L8IJ69	Protein prenyltransferase alpha subunit repeat-containing protein 1	1	1	46338.48	6.53
L8IH73	Uncharacterized protein	3	2	46381.24	9.17
B7XA48	Aspartate aminotransferase	1	1	46411.31	7.09
L8I2J5	Ammonium transporter Rh type A (Fragment)	1	1	46531.64	5.72
L8I9Z4	Adenosylhomocysteinase (Fragment)	3	3	47055.75	5.69
Q52ZH0	Calpastatin type IV	2	2	47125.4	4.7
D2U6Q1	Haptoglobin (Fragment)	8	6	47547.58	8.26
G3N233	Uncharacterized protein	1	1	47895.71	9.63
L8IKB7	26S protease regulatory subunit 7	9	8	48633.3	5.71
P11179	Dihydrolipoyllysine-residue succinyltransferase component of 2-oxoglutarate dehydrogenase complex, mitochondrial	1	1	48971.98	9.1
L8IG31	Uncharacterized protein	1	1	50072.82	6.58
Q3SX22	BSD domain-containing protein 1	1	1	50342.11	4.47
E1BKY9	Uncharacterized protein	3	3	50379.92	8.68
Q2YDN8	Inactive serine/threonine-protein kinase VRK3	1	1	50548.85	8.82
L8IAP6	Rab GDP dissociation inhibitor	2	2	50655.79	6.32
P31754	Uridine 5'-monophosphate synthase	13	11	52228.61	6
L8J599	Serine palmitoyltransferase 1	1	1	52815.51	5.63
L8IWU9	XK-related protein	1	1	53016.1	8.74
Q8WMX8	Ankyrin repeat, SAM and basic leucine zipper domain-containing protein 1	1	1	53183.52	5.76
L8ILF7	Mixed lineage kinase domain-like protein (Fragment)	1	1	53267.92	6.09
G3N3E9	Uncharacterized protein	1	1	53560.96	9.69
L8IDM3	Adenylyl cyclase-associated protein	1	1	53609.62	6.72
F1N2L9	4-trimethylaminobutyraldehyde dehydrogenase	1	1	53990.56	5.84
P58352	Solute carrier family 2, facilitated glucose transporter member 3	2	2	54019.03	5.55
F1MZL6	V-type proton ATPase subunit H	1	1	54089.4	6.18
F1MP10	Uncharacterized protein	1	1	54150.37	6.27
F1N206	Dihydrolipoyl dehydrogenase	1	1	54186.59	7.59
E1BMF2	Uncharacterized protein	1	1	54678.05	7.08
A4IFH5	Alanine aminotransferase 1	1	1	55274.87	7.08
F1MK34	Uncharacterized protein	1	1	55469.44	6.75
Q3SWX3	Aminopeptidase-like 1	1	1	55740.97	6.41
Q0P5A6	26S proteasome non-ATPase regulatory subunit 5	11	10	56041.84	5.2
E1BFN6	Uncharacterized protein	1	1	56302.61	6.37
F1N596	Uncharacterized protein	1	1	57201.78	8.91
Q2YDN4	Coiled-coil domain-containing protein 105	1	1	57642.07	9.84
I6X9J1	Mucin1-cell surface associated protein	1	1	58235.15	6.23
E1B9D9	Uncharacterized protein	1	1	58274.39	7.52
L8J1Y0	Caspase recruitment domain-containing protein 9 (Fragment)	1	1	58931.07	5.57
L8IID4	Coiled-coil domain-containing protein 65	1	1	59440.47	5.91
Q08DY5	Nuclear receptor binding protein 1	1	1	59884.07	5.02
L8IF25	26S proteasome non-ATPase regulatory subunit 3	7	7	60860.79	8.79
L8IGU6	Protein FAM184B (Fragment)	1	1	61751.22	5.91
L8HW81	Glucose-6-phosphate isomerase (Fragment)	4	4	64591.92	8.2
Q3SZI2	Lamin A/C	1	1	65120.95	6.54
Q3ZC32	Optineurin	1	1	65237.76	5.16
A6QPL7	DDN protein	1	1	66093.01	11.11
E1B761	Uncharacterized protein	2	2	67491.08	6.67
Q5EA82	Chromosome 6 open reading frame 11	1	1	67523.59	9.86
Q6B855	Transketolase	6	5	67905.04	7.56
L8J1A5	Zinc finger protein 48	1	1	68557.57	9.41
F1MJJ8	Radixin	5	5	68583.05	5.88
L8IQT8	Stress-induced-phosphoprotein 1 (Fragment)	6	6	68588.08	8.25
E1BL88	Uncharacterized protein	3	2	69566.73	9.46
A6QQ11	PGM2 protein (Fragment)	3	3	69581.06	6.28
L8ITJ0	Protein FAM178B (Fragment)	1	1	70131.67	6.62
E5D619	Heat shock 70 kDa protein 1A	20	17	70271.68	5.68
A7XV32	HSP70	13	11	70393.8	5.49
L8I6M8	Glucose 1,6-bisphosphate synthase	1	1	70490.65	5.94
Q1RMT6	Drebrin 1	1	1	72160.28	4.38
A7YW45	Protein arginine N-methyltransferase 5	1	1	72627.87	5.88
L8IC80	Stress-70 protein, mitochondrial (Fragment)	2	2	73930.94	5.97
L8IEN6	Potassium voltage-gated channel subfamily B member 1 (Fragment)	1	1	74156.15	8.88
L8HYQ4	Coiled-coil domain-containing protein 162 (Fragment)	1	1	75200.66	8.69
L8ILT2	Erythrocyte membrane protein band 4.2 (Fragment)	14	12	77097.73	6.83
B8R1K3	Transferrin	1	1	77657.33	6.92
L8J3D6	DCC-interacting protein 13-alpha (Fragment)	1	1	77671.3	5.4
O77698	Lactotransferrin	1	1	77729.06	8.28
Q2KJ13	Family with sequence similarity 48, member A	2	1	80833.52	8.42
G5E6K2	Uncharacterized protein	1	1	80878.6	9.68
P80227	Acylamino-acid-releasing enzyme	12	11	81092.5	5.18
A1Z1N7	Micromolar calcium-activated neutral protease 1 large subunit	4	4	82143.26	5.48
L8J570	Receptor protein-tyrosine kinase (Fragment)	2	1	83340.25	7.04
A0A088QFM6	Heat shock protein 90kDa alpha	6	5	83362.39	4.98
F1MKQ4	Uncharacterized protein	1	1	85597.02	9.23
L8IDH8	Potassium/sodium hyperpolarization-activated cyclic nucleotide-gated channel 3	1	1	86666.4	9.77
Q08DE9	CUL2 protein	1	1	86955.11	6.46
F6QHJ6	Uncharacterized protein	1	1	87849.22	6.42
E1BKY3	Uncharacterized protein	1	1	88284.54	8.73
Q3ZBT1	Transitional endoplasmic reticulum ATPase	14	12	89328.77	5.13
G3MXC5	Uncharacterized protein	1	1	93542.42	6.17
G5E593	Uncharacterized protein	6	5	95150.44	7.9
L8ISG7	Endoplasmic reticulum metallopeptidase 1 (Fragment)	1	1	95577.48	7.05
L8IQA0	Protein 4.1	38	22	96163.66	5.39
Q6QME8	Protein argonaute-2	5	4	97387.21	9.34
E1BLT9	Uncharacterized protein	1	1	97805.69	8.41
Q5W5U3	Hexokinase 1	1	1	102205.72	6.29
E1BP64	Uncharacterized protein	2	2	102371.41	6.11
F1MC63	Uncharacterized protein	1	1	102852.13	6.07
F1CKK3	Toll-like receptor 3	1	1	103422.64	6.7
A8E655	SLC8A1 protein	1	1	104126.62	4.84
L8ITI3	Zinc finger CCCH domain-containing protein 18	1	1	107112.39	8.93
E1BLV1	Oxysterol-binding protein	1	1	108437.43	5.94
L8IU56	Coiled-coil domain-containing protein 39	1	1	108688.14	6.34
L8IHJ4	Aminopeptidase	1	1	109785.65	5.15
E1BDV7	Uncharacterized protein	1	1	110066.66	6.19
F1MF54	Uncharacterized protein	1	1	110857.38	9.6
L8IPC3	NAD(P) transhydrogenase, mitochondrial	1	1	113908.41	8.4
E1BLH0	Uncharacterized protein	2	2	115627.27	9.23
F1N140	Uncharacterized protein	2	2	118942.45	8.76
L8J0C0	MHC class II transactivator (Fragment)	1	1	119893.61	5.67
F1MJW5	Uncharacterized protein	1	1	125175.95	6.39
L8IJG6	Exportin-7 (Fragment)	1	1	125545.03	5.79
L8I1K4	Ubiquitin carboxyl-terminal hydrolase 7 (Fragment)	1	1	126297.62	5.46
L8J128	Phosphoinositide phospholipase C	1	1	130102.95	5.48
F1MY77	Uncharacterized protein	1	1	131421.8	6.31
L8ICT8	Uncharacterized protein	2	2	137720.45	6.22
Q8MI28	Limbin	1	1	137810.33	6.28
E1BLT5	Uncharacterized protein	1	1	140379.39	8.66
E1BM01	Uncharacterized protein	1	1	143087.6	6.91
G3X7C0	Structural maintenance of chromosomes protein	1	1	143189.14	7.51
L8HUL0	Protein SFI1-like protein	1	1	147525.13	11.49
A5D794	GTPase-activating protein and VPS9 domain-containing protein 1	1	1	157373.68	5.05
E1BPY6	Uncharacterized protein	1	1	161613.97	5.41
L8ILY1	Gem-associated protein 5	1	1	168529.57	6.24
F1MQX9	Centrosomal protein of 290 kDa	2	2	169920.11	6.32
E1BN16	Uncharacterized protein	3	2	169955.57	5.54
L8J612	WD repeat-containing protein 90 (Fragment)	1	1	186968.12	6.5
F1MC50	Uncharacterized protein	1	1	190870.09	8.39
E1BM83	Uncharacterized protein	2	2	194408.55	8.72
L8IHY8	Echinoderm microtubule-associated protein-like 5	1	1	212272.21	8.11
E1BKT4	Uncharacterized protein	1	1	222481.51	6.76
L8HQ97	Myosin-7 (Fragment)	1	1	222518.19	5.7
E1BK77	Uncharacterized protein	1	1	224052.42	6.64
L8I9P1	HEAT repeat-containing protein 5B	1	1	224309.96	6.67
L8HNG2	Retinitis pigmentosa 1-like 1 protein (Fragment)	1	1	226900.61	4.7
E1BGM7	Uncharacterized protein	1	1	235347.36	5.25
L8IGW6	Putative G-protein coupled receptor 179	1	1	242848.97	5.36
G3MZJ0	Uncharacterized protein	1	1	269381.55	5.46
L8HQC4	Zinc finger homeobox protein 2	1	1	270327.95	5.68
F1MIB3	Uncharacterized protein	1	1	283427.6	7.54
E1BIR8	Uncharacterized protein	1	1	297243.5	9.45
G3N1C8	Uncharacterized protein	2	2	492970.78	5.78
E1BED7	Uncharacterized protein	1	1	576028.09	5.96

### Theoretical Two-Dimensional Distribution of the Identified Proteins

**Figure 3:**
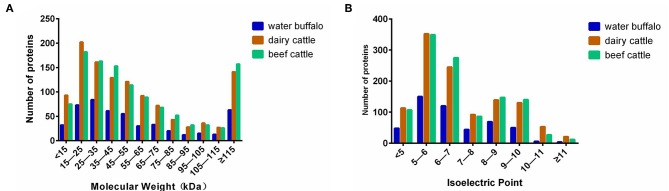
The distributions of the Mw and pI values of the identified erythrocyte proteins of water buffalo, dairy cattle, and beef cattle were comparatively analyzed. That of water buffalo is shown in blue, that of dairy cattle in red, and that of beef cattle in green. **(A)** The distribution of Mw. **(B)** The distribution of pI.

The distributions of the Mw and pI values of the identified proteins from water buffalo, dairy cattle, and beef cattle are shown in Figure 3. Mw and pI were calculated by using the compute Mw/pI tool (http://cn.expasy.org/tools/pi_tool.html) according to the predicted amino acid sequences. They both played directive roles in the characterization of the proteins. Most of the identified proteins of water buffalo, dairy cattle, and beef cattle were in the range of 15 to 55 kDa and more than 115 kDa, accounting for 68.4% (336/491), 65.9% (754/1,145), and 67.3% (769/1,143) of their totals, respectively. Analysis of molecular weight revealed that there was a significant difference in the range of 15 to 25 kDa, with the number of proteins being obviously less in water buffalo than in dairy and beef cattle.

In terms of pI, the great majority of the identified proteins of water buffalo, dairy cattle, and beef cattle were in the range of 5–7, accounting for 54.9% (270/491), 52.1% (597/1,145), and 54.6% (624/1,143) of their totals, respectively. In the range of 5–6, water buffalo had a significantly lower protein count than do dairy and beef cattle.

### Gene Ontology Annotation

**Figure 4:**
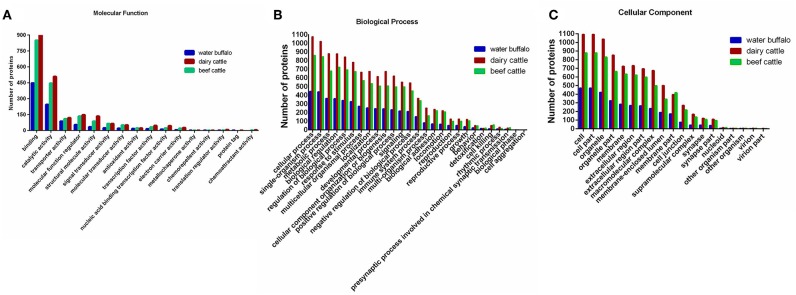
A total of 2,222 identified erythrocyte proteins of water buffalo, dairy cattle, and beef cattle were annotated, respectively, in terms of molecular function, biological process, and cellular component. **(A)** Molecular function. **(B)** Biological process. **(C)** Cellular component. Those of water buffalo are shown in blue, dairy cattle in red, and beef cattle in green.

A total of 2,222 proteins of water buffalo, dairy cattle, and beef cattle were annotated in terms of molecular function, biological process, and cellular component according to the Gene Ontology Annotation (http://www.ebi.ac.uk/goa/) (Figure 4).

For the molecular function annotation, the numbers of water buffalo, beef cattle, and dairy cattle in level two were 15, 15, and 16 respectively, of which 14 were common to them all. A large proportion of proteins in level two were assigned to binding (GO:0005488) and catalytic activity (GO:0003824), significantly more than other categories. The majority of proteins in binding categories were assigned to protein binding (GO:0005515), ion binding (GO:0043167), and organic cyclic compound binding (GO:0097159). Remarkably, the number of proteins in oxygen binding (GO:0019825) was higher in water buffalo than in beef cattle and dairy cattle, even though the number was far lower in other subcategories in level three. This may indicate that oxygen binding is more active in water buffalo, which may be a benefit for *Babesia* survival. Most proteins in catalytic activity were relevant to hydrolase activity (GO:0016787), oxidoreductase activity (GO:0016491), and transferase activity (GO:0016740).

In terms of the biological process categories, most proteins were categorized into metabolic processes (GO:0008152), cellular processes (GO:0009987), and single-organism processes (GO:0044699). Among the GO terms, there was no significant difference in processes between species, even though cell aggregation was exclusive to cattle. It was noteworthy that far fewer proteins were categorized into cellular processes in water buffalo than in dairy cattle and beef cattle, unlike for other processes.

In the cellular component categories, the number of proteins was far higher for dairy cattle than for water buffalo and beef cattle in level two. Most of the proteins were assigned to cell (GO:0005623), cell part (GO:0044464), organelle (GO:0043226), organelle part (GO:0044422), and membrane (GO:0016020). Among these, the numbers of plasma membrane (GO:0005886) components for water buffalo, beef cattle, and dairy cattle were 170, 408, and 403 respectively, of which 92 proteins were unique to water buffalo.

### Significant Differences in Water Buffalo, Dairy Cattle, and Beef Cattle

The number of peptides (peptide count) is directly connected with the relative abundance of the proteins in erythrocytes as identified by LC-MS/MS. Therefore, based on the number of peptides, peptide counts of ≥20 of the identified erythrocyte proteins of water buffalo, beef cattle, and dairy cattle were selected and compared with each other. The number of peptide counts ≥20 were 25, 56, and 50 in water buffalo, beef cattle, and dairy cattle, respectively. Even though the number with a peptide count ≥20 in water buffalo was lower than in beef cattle and dairy cattle, the species of those proteins were similar. Most were hemoglobin, skeleton proteins (spectrin, ankyrin, actin), heat shock proteins, anion exchange protein, and so on. Remarkably, putative gamma-globin and putative epsilon-globin were only detected in water buffalo; they were not detected in beef cattle and dairy cattle. Furthermore, the relative abundance of putative gamma-globin and putative epsilon-globin was high in all of the identified proteins, and the peptide counts were 130 and 76, respectively. Therefore, gamma-globin and epsilon-globin may play key roles and are promising explanations for *B. orientalis* only invading or multiplying in the RBCs of water buffalo.

## Discussion

The hemoprotozoan was identified as a novel *Babesia* species and named *B. orientalis* in 1997 (13). The only natural host was found to be water buffalo, and not beef cattle and dairy cattle, although all of them belong to the tribe of *Bovini* (10, 13). In contrast, *B. bovis* and *B. bigemina* can infect not only water buffalo but also beef cattle and dairy cattle. To date, no studies or articles have become available regarding this difference. This is because many challenges and difficulties limit the investigation of this problem, including the difficulty of obtaining the parasites, the difficulty of continuous cultivation, the non-applicability of gene-editing techniques (CRISPR), and so on.

Due to the fact that *Babesia* can only invade and reside in erythrocytes, the parasite will interact with the erythrocyte through ligands and receptors (33). Many studies have focused on this, and several interaction ligands in parasites and receptors in erythrocytes have been characterized in plasmodium (34). However, there was no significant information available on the recognition ligands and receptors in *Babesia*. Furthermore, most studies pay attention to finding the ligands in the membrane of erythrocytes. However, when parasites reside into the erythrocyte, the contents of the RBC are equally necessary to the parasites. Therefore, this study was from the perspective of the integral erythrocyte proteome including both membrane and cytoplasmic proteins, making it more comprehensive and rigorous. In this study, a comprehensive analysis was performed to compare the erythrocyte proteomes of water buffalo, beef cattle, and dairy cattle. Overall, a total of 491, 1,143, and 1,145 proteins were identified in water buffalo, beef cattle, and dairy cattle, respectively. The number for water buffalo was far less than for beef cattle and dairy cattle, particularly in the range from 15 to 25 kDa, which was also exhibited in the SDS-PAGE results. Furthermore, the erythrocyte protein profile of beef cattle was far more similar to that of dairy cattle, and both were significantly divergent from that of water buffalo. Some significant molecular biases to water buffalo were identified, which may be related to the exclusive survival of *B. orientalis* in the RBCs of water buffalo. Putative gamma-globin and putative epsilon-globin were not detected, and no information is available for beef cattle and dairy cattle to date. Moreover, all of the identified proteins of water buffalo were relatively rich in putative gamma-globin and putative epsilon-globin. The two proteins were encoded by the hemoglobin subunit beta (HBB) gene and have functions in heme binding, iron ion binding, oxygen binding, and oxygen carrier activity. The number of proteins in oxygen binding (GO:0019825) in water buffalo is far higher than in beef cattle and dairy cattle, which increases the significance of deep investigation of these two proteins. Hemoglobin is vital to hemoprotozoan survival inside the RBC and, to date, it is regarded as the main energy source for most of the hemoprotozoan (35). Moreover, hemoglobin is covalently modified in order to inhibit the intake of amino acids by plasmodium but does not affect the normal functions (36). One article has also reported that malaria can cause an imbalance in the globin expression by using the CD34+ haematopoietic stem cell culture system (37). Therefore, further investigation of whether gamma-globin and epsilon-globin are the main reasons for the water buffalo infection specificity of *B. orientalis* would be worthwhile.

All in all, this study is the first to characterize and detail the erythrocyte protein profiles of water buffalo, beef cattle, and dairy cattle by using shotgun technology. In combination with bioinformatics analysis, it has clearly represented the differences among the erythrocyte proteomes of water buffalo, beef cattle, and dairy cattle. Even so, there are many challenges and obstacles that must still be faced. This study was the first to try to find some clues and to explain why water buffalo is the only host of *B. orientalis* and has provided new insights into this question. This study can also act as a guide for the development of vaccines and anti-*B. orientalis* survival agents.

## Conclusion

In conclusion, this study obtained the complete erythrocyte proteomes of water buffalo, beef cattle, and dairy cattle and performed comparative analysis from several aspects including mw, pI, molecular function, biological process, and cellular component. A total of 290 uniquely expressed proteins were identified in water buffalo, which might be related to the infection specificity of *B. orientalis* to water buffalo. The mechanism for infection specificity is complex, and more work needs to be done to elucidate the reasons for the exclusive survival of *B. orientalis* in the erythrocytes of water buffalo rather than in beef and dairy cattle.

## Data Availability Statement

All data obtained in this study had been deposited to the ProteomeXchange Consortium (http://proteomecentral.proteomexchange.org) via the iProX partner repository with the dataset identifiers PXD011408 and IPX0001371000.

## Ethics Statement

The experimental animals were housed and treated in accordance with the stipulated rules for the regulation of the administration of affairs concerning experimental animals of P. R. China. All experiments were performed under the approval of the Laboratory Animals Research Centre of Hubei Province and the Ethics Committee of Huazhong Agricultural University (Permit number: HZAUCA-2016-007).

## Author Contributions

JG performed the experiments. JG and YS participated in the data analysis. JG, YS, and YT helped with the diagnostic assays. JG and JZ edited the manuscript. All authors read and approved the final manuscript.

### Conflict of Interest

The authors declare that the research was conducted in the absence of any commercial or financial relationships that could be construed as a potential conflict of interest.

## Supplementary Material

Supplementary Table 1 The detail results of the mass spectrometry (LC-MS/MS).

## Abbreviations

*B. orientalis*: *Babesia orientalis*
*B. bovis*: *Babesia bovis*
*B. bigemina*: *Babesia bigemina*
HSP: Heat shock protein
MALDI-TOF/MS: Matrix-assisted laser desorption/ionization time-of-flight/mass spectrometer
2-DE/MS: Two-dimensional electrophoresis/mass spectrometer
LC-MS/MS: Liquid chromatography mass spectrometer
RBC: Red blood cell
Ip: Isoelectric point
Mw: Molecular mass
PBS: Phosphate buffered saline
Hp: Haptoglobin
Hb: Hemoglobin
HBB: hemoglobin subunit beta.
